# Evaluation of non-motor symptoms in Parkinson’s disease using multiparametric MRI with the multiplex sequence

**DOI:** 10.3389/fnagi.2025.1602245

**Published:** 2025-07-16

**Authors:** He Sui, Zhanhao Mo, Feng Shi, Qing Zhou, Dan Yu, Jiaqi Wang, Lin Liu

**Affiliations:** ^1^China-Japan Union Hospital of Jilin University, Changchun, China; ^2^Shanghai United Imaging Intelligence Co., Ltd., Shanghai, China; ^3^United Imaging Research Institute of Intelligent Imaging, Beijing, China; ^4^United Imaging Research, Shanghai, China

**Keywords:** Parkinson’s disease, non-motor symptoms, quantitative MRI analysis, brain segmentation, early diagnosis

## Abstract

**Background:**

Non-motor symptoms (NMS) in Parkinson’s disease (PD) often precede motor manifestations and are challenging to detect with conventional MRI. This study investigates the use of the Multi-Flip-Angle and Multi-Echo Gradient Echo Sequence (MULTIPLEX) in MRI to detect previously undetectable microstructural changes in brain tissue associated with NMS in PD.

**Methods:**

A prospective study was conducted on 37 patients diagnosed with PD. Anxiety and depression levels were assessed using the Hamilton Anxiety Scale (HAMA) and Hamilton Depression Scale (HAMD), respectively. MRI techniques, including 3D T1-weighted imaging (3D T1WI) and MULTIPLEX - which encompasses T2*-mapping, T1-mapping, proton density-mapping, and quantitative susceptibility mapping (QSM)—were performed. Brain subregions were automatically segmented using deep learning, and their volume and quantitative parameters were correlated with NMS-related assessment scales using Spearman’s rank correlation coefficient.

**Results:**

Correlations were observed between QSM and T2* values of certain subregions within the left frontal and bilateral temporal lobes and both anxiety and depression (absolute *r*-values ranging from 0.358 to 0.480, *p* < 0.05). Additionally, volume measurements of regions within the bilateral frontal, temporal, and insular lobes exhibited negative correlations with anxiety and depression (absolute *r*-values ranging from 0.354 to 0.658, *p* < 0.05). In T1-mapping and proton density-mapping, no specific brain regions were found to be significantly associated with the NMS of PD under investigation.

**Conclusion:**

Quantitative parameters derived from MULTIPLEX MRI show significant associations with clinical evaluations of NMS in PD. Multiparametric MR neuroimaging may serve as a potential early diagnostic tool for PD.

## Introduction

Parkinson’s disease (PD) is primarily characterized by motor dysfunction; however, the majority of patients also exhibit a range of non-motor symptoms (NMS), including hypotension, anxiety, depression, and executive dysfunction. Notably, these NMS often manifest before the typical motor symptoms. Due to the lack of definitive biomarkers (Bloem et al., 2021; Xie and Hu, 2022), distinguishing NMS from standard age-related manifestations remains challenging, frequently resulting in delayed PD diagnosis and suboptimal treatment (Kalia, 2019; Tolosa et al., 2021).

In PD research, MRI serves as a critical tool for identifying pathological changes. However, conventional MRI techniques often fail to detect subtle structural changes in gray and white matter, thereby limiting their sensitivity to PD-related pathological processes. Quantitative MRI (qMRI) offers a potential solution by enabling non-invasive direct quantification of microstructural alterations in apparently normal brain tissues (Xie and Hu, 2022; Vignando et al., 2022). Reduced T2* values indicate iron deposition, particularly in the substantia nigra, which may impair neuronal function and survival via oxidative stress mechanisms (Ryckewaert et al., 2010). Shortened T1 values not only reflect iron deposition but also suggest neurodegenerative processes, such as neuronal loss and tissue remodeling. Changes in T1 values within the basal ganglia reveal more extensive pathological processes (Baudrexel et al., 2010). These imaging features are interconnected and collectively contribute to disease progression. For instance, iron deposition may exacerbate neuronal damage through oxidative stress, while neuronal loss alters the local microenvironment, influencing T1 and T2* values (You et al., 2015).

Multiparametric quantitative MRI (MP-qMRI) extends this approach by utilizing thresholds or mean values from regions of interest as diagnostic benchmarks in multisite studies (Vignando et al., 2022; Luo and Collingwood, 2022; Cheng et al., 2020). Mounting evidence suggests that MP-qMRI measurements of proton density, T1, and T2* can provide standard distributions of these metrics, enhancing assessments of neurological conditions (Vignando et al., 2022; Cheng et al., 2020). However, lengthy scan durations limit widespread adoption. To address this, recent single-scan techniques that rapidly generate multiparametric quantitative images, such as Magnetic Resonance Fingerprinting (MRF), Quantitative Transient-state Imaging (QTI), and Magnetic Resonance Image Compilation (MAGiC), have been developed (Jiang et al., 2022; Gómez et al., 2020; Bipin Mehta et al., 2019). Building upon these modalities, the Multi-Flip-Angle and Multi-Echo Gradient Echo Sequence (MULTIPLEX) incorporates sequences like Susceptibility Weighted Imaging (SWI), proton density-weighted imaging, and quantitative maps including Quantitative susceptibility mapping (QSM), R2*, T2*, T1-mapping, and proton density-mapping. This allows comprehensive brain mapping in under 6 min, facilitating integration into clinical practice and research (Ye et al., 2022).

Brain structural integrity is notably affected by neurodegenerative disorders such as Alzheimer’s disease, PD, and multiple sclerosis (Sidenkova, 2021). Recent advancements in MRI and image processing now make it feasible to analyze brain measures on a subregional basis, providing more precise insights into aging and disease (Basukala et al., 2021; Sheng et al., 2021; Erdaş and Sümer, 2023). These subregional assessments have demonstrated sufficient sensitivity to discern variations in cognitive performance and have consistently exhibited higher sensitivity compared to other macroscopic structural alterations (Galimzianova et al., 2016). However, manual segmentation introduces inefficiencies and potential inaccuracies. Contemporary advancements in computational medical imaging now enable the application of fully automated techniques, including deep learning, to perform operations such as registration and segmentation seamlessly (Jung et al., 2019).

In the present investigation, the objective is to assess the correlation between whole-brain volume and signal changes in PD patients exhibiting NMS by segmenting and quantitatively analyzing MULTIPLEX MR images. The study further seeks to determine the potential of utilizing MP-qMRI measurements to understand intrinsic disease progression in PD and to inform or oversee initial therapeutic interventions.

## Materials and Methods

### Participant sample

This prospective study was approved by the Medical Research Ethics Committee of China-Japan Union Hospital of Jilin University (No. 2023053014). All procedures involving human participants were conducted in strict accordance with the ethical standards set by the institutional and national research committees, as well as the 1964 Declaration of Helsinki and its amendments. Since this was not a clinical trial, no trial number was assigned. In line with the Declaration of Helsinki, all participants provided written informed consent. Comprehensive physical, neurological, and neuropsychological assessments were conducted on each participant, who met the clinical diagnostic criteria established by the UK PD Brain Bank. Experienced neurologists conducted the clinical evaluations. Motor disability was assessed using the Movement Disorder Society-sponsored revision of the Uni-fied Parkinson’s Disease Rating Scale (MDS-UPDRS) (Goetz et al., 2008), while the Hoehn and Yahr (H&Y) staging was utilized to gauge disease severity (Hoehn and Yahr, 1967). According to the diagnostic guidelines, patients with H&Y stages 1-2 are categorized as early-stage PD patients (generally corresponds to the lower scoring range on the motor subsection of the UPDRS) (Postuma et al., 2015). Cognitive function was evaluated using the Mini-Mental State Examination (MMSE) (Folstein et al., 1983).

**FIGURE 1:**
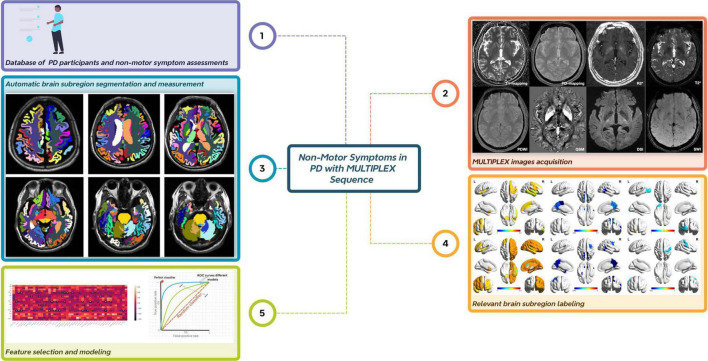
Overall flow chart of the experiment. The entire experimental process comprises five key steps. First, data are collected from Parkinson’s disease patients, and assessments of non-motor symptom scales are performed. Second, image data are acquired using the MULTIPLEX sequence. Third, automatic brain region segmentation and quantitative data measurement are achieved through deep learning technology. Fourth, brain regions associated with the studied non-motor symptoms are identified and labeled. Finally, brain region feature selection is conducted, followed by model performance evaluation.

**FIGURE 2:**
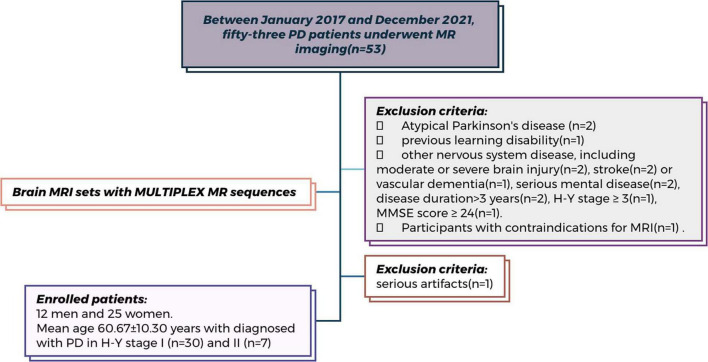
Flowchart of patient enrollment. Flowchart of patient enrollment in this prospective study conducted at China-Japan Union Hospital of Jilin University (Between January 2017 and December 2021). Among the 53 participants initially screened, 16 were excluded based on predefined criteria (detailed reasons are shown in the figure). Therefore, a total of 37 participants underwent MR head MULTIPLEX sequence scanning and were included in the final analysis.

Exclusion criteria included atypical Parkinson’s disease, pre-existing learning disabilities, and other central nervous system pathologies such as moderate to severe traumatic brain injuries, stroke or vascular dementia, and significant psychiatric or medical conditions. Patients with a disease duration exceeding 3 years, an H&Y stage of ≥ 3, or an MMSE rating scale of ≥ 24 were also excluded. Additionally, individuals with contraindications for MRI were not considered. The demographic and clinical characteristics of all PD patients are summarized in Table 1. The experimental workflow is presented in Figure 1, while the patient enrollment process is illustrated in Figure 2.

**TABLE 1:** Participant characteristics.

Clinical characteristics	Mean	SD	Min	Max
12 male,25 female				
Clinically dominate side: 20 right, 7 left				
H&Y Stage	1.72	0.65	3	1
Disease duration (year)	5.49	3.52	1	16
MDS-UPDRS	35.59	16.44	13	65
HAMD	10.86	5.34	4	26
HAMA	12.54	7.02	2	33

H&Y Stage, H&Y Hoehn and Yahr stage; MDS-UPDRS, MDS-UPDRS Movement Disorders Society Unified Parkinson’s Disease Rating Scale; MMSE, Mini-mental State Examination; HAMD, Hamilton Depression Scale; HAMA, Hamilton Anxiety Scale.

### Non-motor symptom assessments

Two experienced movement disorder specialists, LZ (20 years of experience) and YC (15 years of experience), conducted neurological evaluations on the participants. These specialists administered a range of non-motor assessments to the PD patients. Specifically, the Hamilton Anxiety Scale (HAMA) was used to assess and categorize anxiety disorders, while the 17-item Hamilton Rating Scale for Depression (HAMD) was utilized for depression assessment. Patients ceased antiparkinsonian medications at least 12 h prior to these clinical evaluations (Leentjens et al., 2000; Hamilton, 1959).

### MRI acquisition

MRI scans were conducted using a 3-Tesla MRI system (uMR780; United Imaging Healthcare, Shanghai, China) paired with a 32-channel head coil. The MRI protocols included sagittal 3D T1-weighted imaging (3D T1WI), axial T2-weighted imaging (T2WI), axial fluid-attenuated inversion recovery (FLAIR) T2WI, and MULTIPLEX sequences, which comprised T2*-mapping, QSM, T1-mapping, and proton density-mapping. Specific parameters of these sequences detailed in Table 2.

**TABLE 2:** Parameters of MRI sequences.

Parameters	Sagittal 3D T1WI	Axial T2WI	Flair T2WI	Multiplex sequences
FOV (mm)	210 × 210	200	180	190
Resolution (mm^3^)	0.81 × 0.81 × 0.80	0.76 × 0.68 × 6.00	1.12 × 0.90 × 6.00	1.03 × 0.82 × 2.00
TR/TE	7.5/3.2	4,452/120.96	9,000/101.52	35.5/3.05
FA	9	90	90	–
TA (min: s)	06:36	00:22.6	00:54.1	07:15
Slice thickness (mm)	0.8	6	6	2
Slices	–	18	18	–

FOV, field of view; TR, repetition time; TE, echo time; FA, flip angle; TA, acquisition time.

### Segmentation and evaluation of subregions

The deep learning model developed on the United Imaging platform^1^ was employed to automatically segment the whole-brain subregions from each patient’s MULTIPLEX data, including 3D T1WI, T2* mapping, QSM, T1 mapping, and proton density mapping. This process was guided by the Automatic Anatomical Labeling Atlas version 3 (AAL3) (Rolls et al., 2020). The training methodology and reference standards closely aligned with those reported in prior studies (Desikan et al., 2006). The automatic segmentation results encompassed a total of 106 subregions, including 22 temporal lobe structures, 20 frontal lobe structures, 12 parietal lobe structures, 8 occipital lobe structures, 8 cingulate gyrus structures, 2 insular structures, 12 subcortical gray matter structures, as well as cerebral white matter, ventricles, the cerebellum, and other anatomical regions. Notably, left and right hemispheric structures were identified as distinct entities. After automatic segmentation by the deep learning model, two senior radiologists (HS, ZH Mo) with over 10 years of experience reviewed the results. The two radiologists independently verified the accuracy of the segmentation results for all 106 sub-regions in this study. The inter-rater agreement on image segmentation accuracy was assessed using Cohen’s kappa (κ = 0.88).

### Feature selection and modeling

Independent samples *t*-tests were employed to identify features significantly associated with HAMA and HAMD rating scales, retaining those with a *p*-value less than 0.01. To further refine the feature set, the least absolute shrinkage and selection operator (LASSO) regression was applied, performing both variable selection and regularization to enhance predictive accuracy and interpretability. The optimal regularization parameter (lambda) for LASSO was determined using a grid search with 10-fold cross-validation within the training dataset. After feature selection, the remaining features served as input for the classifiers. Logistic regression (LR) models were constructed to predict elevated HAMA and HAMD rating scales based on the selected imaging features.

### Receiver operating characteristic curve analysis and area under the curve calculation

To evaluate the diagnostic accuracy of the LR model in predicting elevated HAMA and HAMD rating scales, we performed ROC curve analysis. The ROC curves were generated by plotting the true positive rate (sensitivity) against the false positive rate (1-specificity) at various threshold levels of the predictor. Additionally, the Area Under the Curve (AUC) was calculated to measure the overall discriminative power. An AUC of 1.0 represents perfect discrimination, while an AUC of 0.5 indicates no better than random guessing.

### Statistical analysis

Statistical analyses were conducted using SPSS Statistics (version 26.0, IBM Corporation, Armonk, NY, United States). Prior to analysis, data normality was assessed using the Shapiro-Wilk test. Spearman’s rank correlation coefficient was used to examine the associations between the volumes and quantitative imaging parameters of each brain subregion and the rating scales on the HAMA and HAMD scales. Statistical significance was set at *p* < 0.05.

## Results

### Participant characteristics

Between January 2017 and December 2021, 37 right-handed patients (12 men and 25 women; mean age 60.67 ± 10.30 years) diagnosed with PD at Hoehn and Yahr stages I (*n* = 30) and II (*n* = 7) were selected for analysis following rigorous inclusion and exclusion criteria. None exhibited indications of atypical Parkinson’s disease or severe cognitive impairment. All participants demonstrated significant positive responses to dopaminergic treatment and underwent examination at their optimal dosages.

In the UPDRS section evaluating motor aspects of daily life, patients registered an average score of 35.59 (SD 16.44, range 12–65). Regarding depression assessment, patients had an average HAMD rating scale of 10.86 (SD 5.34, range 4–26). The aggregate HAMA rating scale for these PD patients was 12.54 (SD 7.02, range 2–33).

### Automatic segmentation results of whole brain subregions

The 106 subregions encompassed the following: temporal lobe (hippocampus, parahippocampal gyrus, amygdala, entorhinal cortex, fusiform gyrus, temporal pole, superior/middle/inferior temporal gyri, transverse temporal gyrus); frontal lobe (precentral cortex, superior frontal gyrus, rostral/caudal middle frontal gyri, frontal pole, lateral/medial orbitofrontal cortices, pars opercularis/orbitalis/triangularis); parietal lobe (postcentral cortex, paracentral lobule, superior/inferior parietal lobules, precuneus, supramarginal gyrus); occipital lobe (cuneus, lingual gyrus, pericalcarine cortex, lateral occipital cortex); cingulate gyrus (anterior/mid/posterior cingulate gyri, isthmus of cingulate gyrus); insular lobe; subcortical gray matter (caudate, putamen, pallidum, thalamus, nucleus accumbens, claustrum); cerebral white matter; ventricles (lateral, third, fourth ventricles, cerebrospinal fluid); cerebellar structures (cortex, white matter); and additional regions (choroid plexus, inferior horn of lateral ventricle, brainstem, optic chiasm, corpus callosum). Notably, left and right hemispheric structures were identified as distinct entities.

### Volume of brain subregions correlate with non-motor symptoms in PD

**FIGURE 3:**
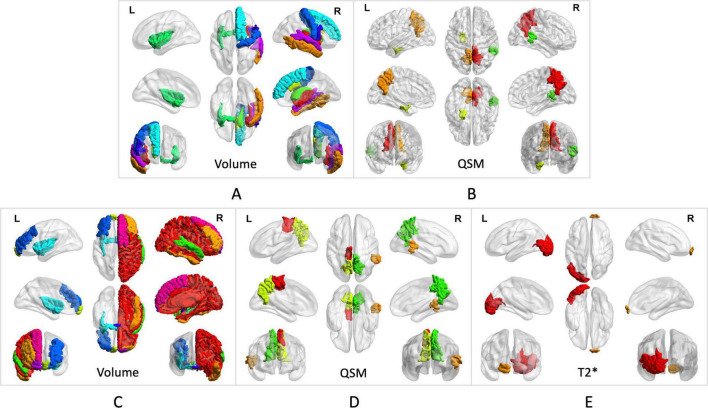
Brain subregion correlates with anxiety in PD. **(A)** The volumes of the right superior frontal gyrus, middle cingulate cortex, caudate nucleus, middle temporal gyrus, and bilateral insular lobe demonstrated negative associations with the HAMA rating scales. **(B)** QSM values from the right superior temporal gyrus, bilateral precuneus, and left entorhinal cortex showed negative correlations with the HAMA rating scales. Brain subregion correlates with depression in PD. **(C)** The volumes of the left anterior middle frontal gyrus, frontal pole, bilateral insular lobe, and right superior temporal gyrus showed a negative correlation with the HAMD rating scales The volume of the right middle cingulate cortex and middle temporal gyrus showed strong correlations with the HAMD rating scales. **(D)** QSM values in the left paracentral lobule, bilateral precuneus, and right superior temporal gyrus showed negative correlations with HAMD rating scales. **(E)** T2* values in the right frontal pole and left lateral occipital gyrus were associated with HAMD rating scales.

The investigation revealed a negative correlation between the volume of the left anterior part of the middle frontal gyrus, frontal pole, bilateral insular lobe, and right superior temporal gyrus and the HAMD rating scale. Strong correlations with the HAMD rating scale were also identified for the volume of the right middle cingulate cortex and middle temporal gyrus. Moreover, the volumes of the right superior frontal gyrus, middle cingulate cortex, caudate nucleus, middle temporal gyrus, and the bilateral insular lobe demonstrated negative associations with the HAMA rating scale (*p* < 0.05). Details are provided in Figures 3A,C and Table 3.

**TABLE 3:** Correlations between volume of related brain subregions and non-motor symptoms of PD patients in MTP sequences estimated by Spearman’s correlation test.

Clinical target	Subregion	*r*	*p*	95% CI
HAMD	R superior frontal gyrus	-0.365	0.037	−0.636 to −0.015
	L anterior part of the middle frontal gyrus	-0.404	0.020	−0.662 to −0.060
	R anterior part of the middle frontal gyrus	-0.358	0.041	−0.631 to −0.006
	R caudal part of middle frontal gyrus	-0.354	0.043	-0.628 to -0.001
	L frontal pole	-0.457	0.008	−0.697 to −0.124
	L insular lobe	-0.420	0.015	−0.673 to −0.079
	R insular lobe	-0.451	0.008	−0.693 to −0.117
	R middle cingulate cortex	-0.658	0.000	−0.820 to −0.398
	R entorhinal cortex	-0.357	0.042	−0.630 to −0.004
	R superior temporal gyrus	-0.456	0.008	−0.697 to −0.123
	R middle temporal gyrus	-0.560	0.001	−0.762 to −0.258
	optic chiasma	-0.377	0.030	−0.644 to −0.028
HAMA	R precentral gyrus	-0.388	0.026	−0.651 to −0.041
	R superior frontal gyrus	-0.406	0.019	−0.663 to −0.062
	R tegmentum of pons	-0.363	0.038	−0.634 to −0.012
	L insular lobe	-0.413	0.017	−0.669 to −0.071
	R insular lobe	-0.508	0.003	−0.730 to −0.190
	R middle cingulate cortex	-0.446	0.009	−0.690 to −0.111
	R caudate nucleus	-0.417	0.016	−0.671 to −0.075
	R superior temporal gyrus	-0.361	0.039	−0.632 to −0.009
	R middle temporal gyrus	-0.480	0.005	−0.712 to −0.154

CI, confidence interval; L, left; R, right.

### Quantitative indicators of brain subregion correlate with anxiety in PD

**FIGURE 4:**
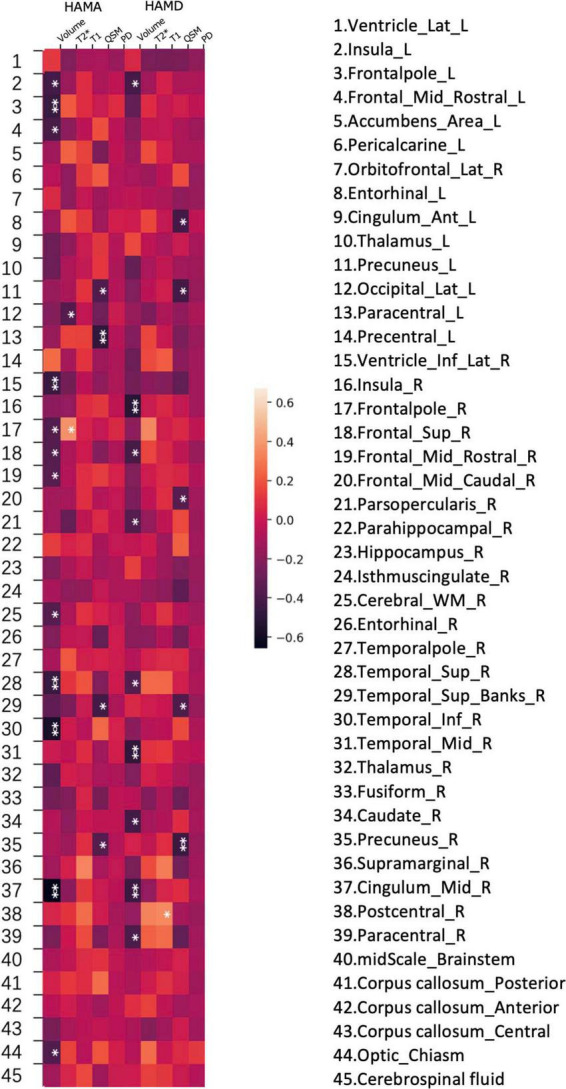
Heatmap of the correlations between various brain subregion indicators and non- motor symptoms in PD. A heatmap is shown demonstrating Spearman correlations between different values (volume, PD (proton density), QSM, T1 and T2*) and HAMD, HAMA rating scales at various regions of the brain.

QSM values from the right superior temporal gyrus, bilateral precuneus, and left entorhinal cortex demonstrated negative correlations with HAMA (*p* < 0.05). These findings are further detailed in Figures 3B, 4, as well as Table 4.

**TABLE 4:** Correlations between values in various brain subregions and non-motor symptoms of PD patients in MTP sequences estimated by Spearman’s correlation test.

Clinical target	Subregion	Parameter	*r*	*p*	95% CI
HAMD	L paracentral lobule	QSM	-0.480	0.005	−0.712 to −0.153
	L precuneus	QSM	-0.376	0.031	−0.643 to −0.027
	R precuneus	QSM	-0.358	0.041	−0.631 to −0.006
	R superior temporal gyrus	QSM	-0.416	0.016	−0.670 to −0.074
	R frontal pole	T2*	0.376	0.031	0.027 to 0.643
	L gyri occipitales laterales	T2*	-0.371	0.033	−0.640 to −0.021
HAMA	R posterior central gyrus	T1	0.349	0.047	−0.005 to 0.624
	R caudal part of middle frontal gyrus	QSM	-0.352	0.044	−0.627 to 0.000
	L entorhinal cortex	QSM	-0.415	0.016	−0.669 to −0.073
	L precuneus	QSM	-0.422	0.015	−0.674 to −0.081
	R precuneus	QSM	-0.450	0.009	−0.693 to −0.116
	R superior temporal gyrus	QSM	-0.386	0.027	−0.650 to −0.039

CI, confidence interval; L, left; R, right; T2*: T2 star.

### Quantitative indicators of brain subregion correlate with depression in PD

In the analyzed PD patients, correlations were observed between HAMD and both T2* and QSM values in specific brain subregions. Specifically, QSM values in the left paracentral lobule, bilateral precuneus, and right superior temporal gyrus exhibited negative correlations with HAMD (*p* < 0.05). Furthermore, T2* values in the right frontal pole and left lateral occipital gyrus demonstrated associations with HAMD (*p* < 0.05). Detailed findings are presented in Figures 3D,E, 4.

### HAMD and HAMA rating scale models

**FIGURE 5:**
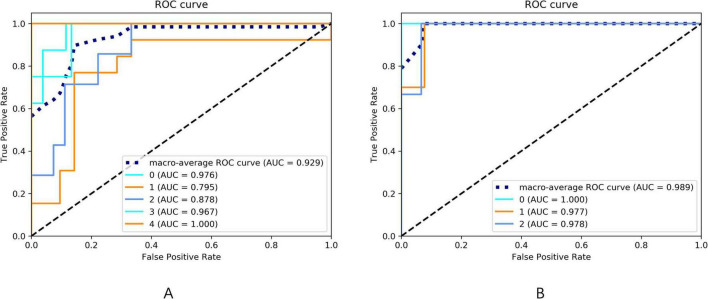
**(A)** The ROC curve of the HAMA rating scale prediction model. The AUC of the ROC curves for the 0–4 five-category rating scale prediction model of HAMA are 0.976, 0.795, 0.878, 0.967, and 1.000, respectively. The macro-average AUC of the ROC curve for the HAMA rating scale prediction model is 0.929. **(B)** The ROC curve of the HAMD rating scale prediction model. The AUC of the ROC curves for the 0–2 three-category rating scale prediction model of HAMD are 1.000, 0.977, and 0.978, respectively. The macro-average AUC of the ROC curve for the HAMD rating scale prediction model is 0.989.

Following the feature selection process, several parameters were identified for constructing the HAMD rating scale prediction model: QSM of the left paracentral lobule and bilateral precuneus, volumes of the middle cingulate gyrus, middle temporal gyrus, and right middle frontal gyrus, T2* of the left frontal pole, and T1 of the left lateral occipital gyrus. For the HAMA rating scale prediction model, the selected features included QSM of the right middle frontal gyrus, left entorhinal cortex, and right precuneus, along with the volumes of the insula, middle cingulate gyrus, caudate nucleus, and right middle temporal gyrus. The retained features after t-tests and LASSO selection are presented in Supplementary Table 1. The performance metrics of these models are presented in Table 5 and Figure 5.

**TABLE 5:** The performance in different models.

Models	SPE	SEN	Youden index	ACC	AUC
HAMD	0.87	0.96	0.83	0.94	0.98
HAMA	0.75	0.91	0.66	0.68	0.92

SPE, specificity; SEN, sensitive; ACC, accuracy; AUC, Area Under Curve.

## Discussion

This study utilized MP-qMRI MULTIPLEX imaging (including 3D T1WI, QSM, T2*-mapping, proton density-mapping, and T1-mapping) in PD patients with NMS. By integrating deep learning-driven automatic brain segmentation and measurement methodologies, we determined that changes in volume and quantitative parameters within brain subregions are discernible. Further analysis revealed correlations between the derived tissue parameter values and NMS manifestations in PD, such as anxiety and depression. These findings underscore the potential of these morphological metrics in early PD, aiding in the preliminary recognition and diagnosis of the disease.

Our analysis indicates that NMS, as assessed with HAMD and HAMA, correlates with atrophy predominantly observed in the frontal, temporal, and parietal regions. These findings align with those documented in early PD patients (Danti et al., 2015; Pereira et al., 2014). In studies of brain structure in psychogenic non-epileptic seizures (PNES), similar conclusions were drawn, noting an association between atrophy in premotor regions and elevated depression rating scales. Conversely, in recently diagnosed PD patients without cognitive impairment, no significant thinning of the parietal or temporal lobes was discerned when compared to control groups (Noh et al., 2014). This discrepancy might be attributed to the heightened sensitivity of our methodological approach, which accentuates nuanced variations in cortical thickness. A pronounced correlation was also identified between insular atrophy and symptoms of depression and anxiety, mirroring findings from studies on traumatic brain injury with PNES (Sharma et al., 2022).

Recent evidence indicates that iron shortens the T2* value and reduces signal in gradient echo amplitude imaging; as a result, iron content is indirectly assessed (Rispoli et al., 2018). QSM is an emergent post-processing technique designed for the quantitative evaluation of tissue magnetization rates (Thomas et al., 2020). Both QSM and T2* are proposed to possess a heightened sensitivity for detecting iron quantities in deep cerebral nuclei. The association of QSM and T2* values in specific regions of the frontotemporal cortex with the cognitive, depression, and anxiety symptoms of PD patients was corroborated by findings from the study conducted by Thomas et al. (2020). Interestingly, traditional iron deposition areas in PD, such as the substantia nigra and red nucleus, were absent in the current results. This may suggest that these neural regions are more intricately tied to motor symptoms rather than non-motor symptoms. A parallel observation was made by Shin et al., where the severity of non-motor symptoms in early PD patients showed no correlation with iron accumulation. This assertion, however, requires validation through extensive future investigations (Chaewon et al., 2018).

Previous studies suggest that the quantitative T1 value might serve as a significant indicator for tracking progressive neuronal loss associated with PD. Given that the T1 value for gray matter typically surpasses that of white matter, PD is associated with a loss of gray matter (Wansapura et al., 1999). In Spearman correlation analysis, a 95% confidence interval (CI) that includes zero indicates a lack of statistical significance in the observed correlation. This implies that the data do not provide sufficient evidence to reject the null hypothesis, as the probability of observing such a correlation—or an even more extreme one—exceeds 5%. Consequently, in our prediction model for brain regions associated with PD-related anxiety, we excluded the T1 value of the right posterior central gyrus (95% CI: −0.005 to 0.624) and the QSM value of the right caudal part of the middle frontal gyrus (95% CI: −0.627 to 0.000), given that neither demonstrated a statistically meaningful association with anxiety symptoms in PD. Thus, no significant association was found between the T1 values of specific brain regions and NMS (anxiety and depression) of Parkinson’s disease in this experiment. Although previous studies have shown that deep gray matter regions (such as the substantia nigra) in Parkinson’s disease patients are significantly affected in the early stage of the disease, with their T1 values shortening three times within 6.5 years compared to the control group, reflecting the loss of gray matter in the contralateral limb, this method may not be effective in evaluating brain regions related to NMS (Nürnberger et al., 2017).

Another key advantage of this study is the integration of deep learning models for multi-region brain structure segmentation, which significantly enhances the objectivity and reproducibility of the research. Compared with traditional manual or semi-automated segmentation approaches, deep learning models offer greater automation and superior feature extraction capabilities, enabling more accurate identification and differentiation of subtle structural variations across distinct brain regions. This high-precision segmentation minimizes human-induced bias and markedly improves the consistency and stability of data processing, thereby enhancing the scientific validity and reliability of the findings. Moreover, applying this model to multi-region brain analysis allows for the simultaneous evaluation of multiple PD-related brain areas, rather than focusing solely on isolated regions. This comprehensive approach facilitates a broader understanding of the disease’s pathophysiological mechanisms across the entire brain, particularly in relation to NMS. For example, PD patients frequently experience non-motor manifestations such as anxiety, depression, and cognitive dysfunction, which are typically mediated by complex interactions among distributed neural circuits (Jiang, 2022). By systematically analyzing these interconnected brain regions using deep learning techniques, we can gain deeper insights into the underlying neural circuitry associated with NMS. Looking ahead, this method can be further applied to conduct more detailed and systematic investigations of additional PD-related non-motor symptoms, ultimately contributing to the construction of a more comprehensive brain structure-function correlation map. Such advancements will not only deepen our understanding of the overall pathological progression of PD but also provide novel strategies and technical support for early clinical diagnosis and personalized therapeutic interventions. Furthermore, as the model continues to evolve and is trained on increasingly diverse datasets, its applicability across larger cohorts and various PD subtypes can be validated, laying a solid foundation for future large-scale clinical studies and translational applications.

This study presents several limitations. Firstly, the limited participant count restricts the broad applicability of the findings. Secondly, the diagnosis of PD relied solely on clinical criteria without pathological confirmation, which might affect the outcome. Lastly, given the potential for participants in this age bracket to possess other neurodegenerative diseases, future expansion in the patient sample size is essential, accompanied by meticulous comparisons across varied age groups.

## Conclusion

This research demonstrates the potential of qMRI MULTIPLEX neuroimaging across the entire brain for examining crucial clinical indices of NMS in PD. PD exhibits an array of microstructural alterations, potentially linked to corresponding pathological and maladaptive processes inherent in the disease’s pathophysiology. The methodologies employed here pave the way for *in vivo* exploration of diverse facets of PD pathology, potentially serving as early diagnostic tools, biomarkers, and parameters to evaluate treatment efficacy in forthcoming studies.

## Data Availability

The raw data supporting the conclusions of this article will be made available by the authors, without undue reservation.

## Footnotes

1
https://urp.united-imaging.com/#/
