# Supplementary material for: Evaluation of non-motor symptoms in Parkinson’s disease using multiparametric MRI with the multiplex sequence
Source: Front Aging Neurosci. 2025 Jul 16;17:1602245. doi: 10.3389/fnagi.2025.1602245 (PMC12307406; doi:10.3389/fnagi.2025.1602245)
Supplement: Supplementary file 1 [file Data_Sheet_1.docx]

**Supplementary Table 1 Retained Features after t-Tests and LASSO Selection**

| ***Clinical Target*** | ***Features retained post t-tests*** | ***Features retained post LASSO selection*** |
| --- | --- | --- |
| HAMD | 18 | 9 |
| HAMA | 15 | 7 |
